# Dosimetric Selection for Helical Tomotherapy Based Stereotactic Ablative Radiotherapy for Early-Stage Non-Small Cell Lung Cancer or Lung Metastases

**DOI:** 10.1371/journal.pone.0035809

**Published:** 2012-04-25

**Authors:** Alexander Chi, Zhongxing Liao, Nam P. Nguyen, Jiahong Xu, James S. Welsh, Si Young Jang, Carol Howe, Ritsuko Komaki

**Affiliations:** 1 Department of Radiation Oncology, West Virginia University, Morgantown, West Virginia, United States of America; 2 Department of Radiation Oncology, University of Texas MD Anderson Cancer Center, Houston, Texas, United States of America; 3 Westat-an Employee-Owned Research Corporation, Rockville, Maryland, United States of America; 4 Department of Radiation Oncology, Louisiana State University, Shreveport, Louisiana, United States of America; 5 Medical Library, University of Arizona, Tucson, Arizona, United States of America; Dresden University of Technology, Germany

## Abstract

**Background:**

No selection criteria for helical tomotherapy (HT) based stereotactic ablative radiotherapy (SABR) to treat early stage non-small cell lung cancer (NSCLC) or solitary lung metastases has been established. In this study, we investigate the dosimetric selection criteria for HT based SABR delivering 70 Gy in 10 fractions to avoid severe toxicity in the treatment of centrally located lesions when adequate target dose coverage is desired.

**Materials and Methods:**

78 HT-SABR plans for solitary lung lesions were created to prescribe 70 Gy in 10 fractions to the planning target volume (PTV). The PTV was set to have ≥95% PTV receiving 70 Gy in each case. The cases for which dose constraints for ≥1 OAR could not be met without compromising the target dose coverage were compared with cases for which all target and OAR dose constraints were met.

**Results:**

There were 23 central lesions for which OAR dose constraints could not be met without compromising PTV dose coverage. Comparing to cases for which optimal HT-based SABR plans were generated, they were associated with larger tumor size (5.72±1.96 cm vs. 3.74±1.49 cm, *p*<0.0001), higher lung dose, increased number of immediately adjacent OARs ( 3.45±1.34 vs. 1.66±0.81, *p*<0.0001), and shorter distance to the closest OARs (GTV: 0.26±0.22 cm vs. 0.88±0.54 cm, *p*<0.0001; PTV 0.19±0.18 cm vs. 0.48±0.36 cm, *p* = 0.0001).

**Conclusion:**

Delivery of 70 Gy in 10 fractions with HT to meet all the given OAR and PTV dose constraints are most likely when the following parameters are met: lung lesions ≤3.78 cm (11.98 cc), ≤2 immediately adjacent OARs which are ≥0.45 cm from the gross lesion and ≥0.21 cm from the PTV.

## Introduction

Helical Tomotherapy (HT) is a technology that delivers fan-beam intensity-modulated radiotherapy (IMRT) under megavoltage computed tomography (MVCT) guidance with continuous and synchronous gantry rotation and couch movement during radiation delivery [1]. Image guided IMRT delivered through HT has been shown to be able to generate highly conformal dose distribution at various anatomical sites since its clinical adaptation [2]. When compared with other techniques of radiation delivery, such as three dimensional conformal radiotherapy (3D-CRT) and conventional linac based IMRT, it may generate superior normal tissue sparing and target dose homogeneity as shown in some studies [3]–[5]. Therefore, it may provide a dosimetric advantage in the sparing of critical organs at risk (OARs) in complex cases, such as the delivery of stereotactic ablative radiotherapy (SABR) in the treatment of centrally located early stage non-small cell lung cancer (NSCLC) or solitary lung metastases from other primaries because of their ability to generate highly conformal dose avoidance of the OARs. This is of key clinical importance in the avoidance of severe toxicities associated with SABR (also called stereotactic body radiation therapy, SBRT), because such toxicities are mostly associated with central location and close proximity to critical structures in the thorax [6]–[10].

We have previously demonstrated the feasibility of HT-based SABR for the treatment of centrally located lung lesions [11]. However, no guidelines for the selection of optimal candidates for this procedure was found after a systematic, and extensive search of the literature on SABR through the PubMed, and Google scholar search engines. This prompted us to investigate the dosimetric selection criteria for HT-based SABR in patients with solitary early-stage NSCLC or metastatic lung tumors using the most common dose fractionation schedule used in our institution, 7 Gy×10 fractions, which we chose due to the previously reported excellent local control (>90%) and minimal toxicity associated with this regimen even for large tumors [12]. A minimal biologically effective dose of 100 Gy_10_ or higher is required for optimal local control [13]. The BED corresponding to this fractionation schedule is 119 Gy_10_. This study will provide preliminary guidelines in the selection of centrally located early stage NSCLC and solitary lung metastases for designing future prospective clinical studies on HT-based SABR in the thorax.

## Materials and Methods

### Patient and tumor characteristics

This study has been approved by the institutional review borad (IRB) at the University of Arizona. Since no actual human subjects was involved, no informed consent was needed per IRB. A total of seventy eight patients who underwent radiation therapy for stage I-II NSCLC, isolated recurrences from a lung primary, or metastases to the lung from other primaries in the department of Radiation Oncology at the University of Arizona from 2005 to 2011 have been included in this study. We retrieved the previous planning CT's for each patient to outline the gross tumor. Among them, 58 centrally located lesions were identified. Central location is defined as the area within 2 cm of the proximal bronchial tree, which includes the lower trachea, carina, mainstem bronchi, and the lobar bronchi. The critical structures are the esophagus, the heart, the spinal cord, major blood vessels, the distal trachea, and the proximal bronchial tree in the majority of the cases. Rarely, the brachial plexus, and the stomach were also in the vicinity of the gross disease.

### Target volume delineation and SBRT treatment planning

All the target delineation was performed in the Pinnacle treatment planning system (Philips Medical Systems, Bothell, WA). Afterwards, each patient's planning CT scan and the contours were transferred into the Helical Tomotherapy planning system (Tomotherapy Inc.) for treatment planning. The planning target volume (PTV) was the clinical target volume (CTV) with a 5 mm expansion to account for set up errors and residual tumor motion. The CTV equals to the gross tumor volume (GTV) and its immediately adjacent areas which are felt to be at high risk for microscopic disease extension. The lungs, esophagus, spinal cord, and the heart were contoured for each patient. The major vessels, major airway and other additional structures were contoured only when they were adjacent to the GTV.

Treatment plans were generated in the Tomotherapy Hi-Art planning system using 6 MV photons delivered without a flattening filter. A binary multi-leaf collimator (MLC) with a leaf width that projects to a 6.25 mm width at the isocenter which is 85 cm away from the X-ray photon source. In the plans, longitudinal aperture sizes of 1.05 cm or 2.5 cm, and pitch of 0.3 were used. The nominal dose rate at the isocenter was 870 cGy/min (SAD). A modulation factor of 3 was set at the beginning of the optimization process. All SBRT plans prescribed 70 Gy delivered in 10 daily fractions to the PTV with heterogeneity corrections using the superposition-convolution algorithm.

All plans were optimized to have at least 95% of the PTV receiving 100% of the prescription dose, which is in accordance with the American society of therapeutic radiation oncology (ASTRO)'s white paper [14]. The dose volume constraints used at our institution are shown in Table 1, which are described in more detail in our previous publication [11]. These parameters (10 fractions) approximate those used in the RTOG 0236 (3 fractions) [15] in their biologically equivalent isodose effect, which were calculated with the linear quadratic formulism using an α/β ratio of 3. An α/β ratio of 2 and the likelihood for intrafractional patient motion was also taken into consideration while deriving a reasonable dose constraint for the spinal cord. Maximum point dose constraints were used as in line with those used in the RTOG 0236, because the majority of the thoracic OARs other than the lungs were serial structures, severe damage to even a small point could be catastrophic as that reported by Onimaru *et al* [9], [16]. Target volume coverage took priority over the dose constraints for the OARs in each case because of the concern for significant decrease in tumor control probability (TCP) when there are significant subvolumes of cold spots [17].

**Table 1:** Dose constraints for the prescription dose of 70 Gy delivered in 10 fractions.

Critical structures	Maximum tolerated dose (Gy)
Spinal Cord*	28
Esophagus	44
Major airway	49
Heart	49
Brachial plexus	38.5
Major vessels	49
Stomach	44
**Total lung** †	
V_20_	20
MLD	9.5

* 30 Gy acceptable in selected cases.

† Total lung volume = total volume of both lungs minus that of the GTV.

V_20_ is the % of the volume receiving 20 Gy. MLD: mean lung dose.

### Data analysis

The size and location of the GTV (and PTV); the number of adjacent critical structures within 2 cm from the edge of the GTV; and the distances of the GTV and PTV to each of these adjacent structures, respectively, were recorded for each patient. These parameters, and the doses to the PTV & the OARs for patients whose treatment plans met the given dose constraints were compared to those from patients whose treatment plans did not met these constraints using the t-test.

## Results

The PTV coverage criteria of ≥95% of the PTV receiving 70 Gy (%PTV_70 Gy_) was met by all 78 treatment plans. Among them, dose constraints for the OARs could not be all satisfied in order to maintain adequate PTV coverage for 23 centrally located lesions (Central_no_). The dose covering 95% of the PTV (D_95_), %PTV_70 Gy_, and the maximum dose to the PTV (PTV_max_) for this group of lesions were compared with those for 35 central (Central) and 20 peripheral lesions (Peripheral) for which the HT SABR plans met all the PTV and OAR dose parameters. The findings are summarized in Table 2. The PTV_max_ was significantly higher, while the D_95_ and %PTV_70 Gy_ were significantly lower in the Central_no_ group.

**Table 2:** Dose Coverage of the PTV for the three groups of lung lesions.

	Mean (Std. Dev.)			*p* value		
	Central	Central_no_	Peripheral	Central vs. Central_no_	Central vs. Peripheral	Central_no_ vs. Peripheral
D_95_ (Gy)	70.60 (0.55)	70.30 (0.50)	70.70 (0.53)	0.0301	0.5587	0.0145
%PTV_70 Gy_	96.90 (1.19)	95.40 (0.43)	97.90 (0.62)	<0.0001	0.0002	<0.0001
PTV_max_ (Gy)	79.10 (4.30)	85.00 (4.01)	75.80 (1.95)	<0.0001	0.0003	<0.0001

PTV: planning target volume. OARs: Organs at risk. D_95_: dose covering 95% of the PTV; %PTV_70 Gy_: percentage of the PTV volume receiving 70 Gy; PTV_max_: maximum dose to the PTV. Std. Dev.: standard deviation; Central: central lesions for which all the dose constraints were met; Central_no_: central lesions for which ≥1 dose constraints were not met; Peripheral: peripheral lesions.

### Dose to the lungs

The dose to the lungs is mainly evaluated by the dose to the total lung (volume_left lung_+volume_right lung_−GTV). There was no statistically significant difference in the volumes of the total lung, the ipsilateral lung, and the contralateral lung among the three groups of lesions (*p*>0.05). The commonly used parameters of the mean lung dose (MLD), and the volume of the total lung receiving 5 Gy, 10 Gy, and 20 Gy (V_5_, V_10_, and V_20_) are listed in Table 3. The V_20_ was kept to below 20% for all three types of lesions. The MLD for the total lung appears to be higher in the Central_no_ group. However, it was below the MLD constraint for most cases in this group.

**Table 3:** Dose parameters for the normal lung tissue.

	Mean (Std. Dev.)			*P* value		
	Central	Central_no_	Peripheral	Central vs. Central_no_	Central vs. Peripheral	Central_no_ vs. Peripheral
**Total lung**						
V_5_	20.6 (4.49)	28.8 (13.4)	21.3 (2.67)	0.0085	0.4843	0.0139
V_10_	14.2 (3.71)	18.5 (10.9)	13.5 (2.94)	0.0775	0.4961	0.0444
V_20_	9.21 (3.48)	10.6 (6.17)	7.21 (2.91)	0.3342	0.0345	0.0251
MLD (Gy)	5.80 (1.71)	6.96 (3.61)	5.10 (1.19)	0.1607	0.1129	0.0273
**Ipsilateral lung**						
V_5_	33.3 (10.6)	39.3 (13.7)	32.4 (6.79)	0.0665	0.6975	0.0400
V_10_	27.1 (8.78)	31.3 (12.7)	24.0 (5.55)	0.1465	0.1164	0.0191
V_20_	18.1 (7.50)	20.7 (11.8)	12.8 (4.88)	0.3621	0.0062	0.0063
MLD (Gy)	9.64 (3.29)	11.5 (5.74)	7.59 (1.89)	0.1605	0.0049	0.0043
**Contralateral lung**						
V_5_	8.53 (6.28)	17.5 (14.9)	8.34 (7.57)	0.0105	0.9197	0.0136
V_10_	1.82 (2.52)	6.09 (10.9)	1.20 (2.18)	0.0847	0.3971	0.0520
V_20_	0.48 (0.73)	1.33 (2.24)	0.53 (1.03)	0.1487	0.9020	0.5006
MLD (Gy)	1.71 (0.60)	2.69 (1.93)	1.74 (0.37)	0.0261	0.8272	0.0291

Std. Dev.: standard deviation. Central: central lesions for which all the dose constraints were met. Central_no_: central lesions for which ≥1 dose constraints were not met. Peripheral: peripheral lesions. V_5_, V_10_, and V_20_: percentage of volume receiving 5, 10, and 20 Gy, respectively. MLD: mean lung dose.

The MLD, V_5_, V_10_, and V_20_ for the ipsilateral and the contralateral lungs are also shown in Table 3 to explore the degree of contralateral lung sparing in HT-based SABR. All three types of lesions were found to have significantly lower doses to the contralateral lung comparing to that to the ipsilateral lung (*p*<0.0001). Worth mentioning is that the MLD and V_20_ for the ipsilateral lung are significantly higher for all the central lesions, and the contralateral lung's MLD and V_5_ are found to be higher in the Central_no_ group when compared to the other two groups.

### Dose to the other OARs

The maximum dose received by the spinal cord, the esophagus, the heart, the major airways, and the major vessels is summarized in Table 4. The Central_no_ group was found to have significantly higher doses for all OARs when compared to other two groups of lung lesions.

**Table 4:** Maximum dose to the organs at risk (including organs immediately adjacent to the tumor).

	Mean (Std. Dev.)			*P* value		
	Central (Gy)	Central_no_ (Gy)	Peripheral (Gy)	Central vs. Central_no_	Central vs. Peripheral	Central_no_ vs. Peripheral
**Spinal cord**	18.6 (6.75)	23.3 (6.58)	15.8 (4.58)	0.0102	0.1126	0.0001
**Esophagus**	21.4 (11.0)	37.1 (16.3)	14.5 (5.33)	0.0003	0.0027	<0.0001
**Heart**	17.2 (19.8)	31.2 (23.9)	10.5 (13.8)	0.0194	0.1856	0.0011
**Major vessels**	45.6 (3.98)	68.0 (8.78)	-	<0.0001	-	-
**Major airway**	36.9 (13.0)	67.4 (10.6)	-	<0.0001	-	-

Std. Dev.: standard deviation. Central: central lesions for which all the dose constraints were met. Central_no_: central lesions for which ≥1 dose constraints were not met. Peripheral: peripheral lesions. Gy: gray.

### Factors influencing the feasibility of HT-based SBRT

A set of tumor factors were investigated to characterize the lesions in the Central_no_ group when compared with the other two groups of lesions. The findings are summarized in Table 5. The tumor size for the Central_no_ group was significantly larger. These central lesions, for which HT-based SABR is not feasible, had more than 2 critical structures immediately adjacent to the GTV. In addition, they were significantly closer to the critical structures with an average OAR to GTV, and PTV distances of 0.26 cm, and 0.19 cm, respectively. The median distribution of our data suggests the most ideal candidates for HT-based SABR should have GTV≤3. 78 cm, or 11.98 cc; PTV≤4.90 cm, or 34.43 cc; ≤2 separate adjacent structures immediately adjacent to the GTV, the minimum GTV to OAR distance of ≥0.45 cm, and the minimum PTV to OAR distance of ≥0.21 cm.

**Table 5:** Comparison of the tumor characteristics of the Central_no_ group of lesions with the Central and Peripheral groups of lesions.

	Central+Peripheral	Central_no_	*P* value
	Mean (Std. dev.)	Mean (Std. dev.)	
GTV (cm)	3.74 (1.49)	5.72 (1.96)	<.0001
GTV_cc	16.4 (21.5)	51.1 (54.4)	0.0065
PTV (cm)	4.90 (1.48)	6.71 (1.97)	<.0001
PTV_cc	43.2 (38.9)	88.7 (74.7)	0.0100
# of separate group of OARs	1.66 (0.81)	3.45 (1.34)	<.0001
Distance to adjusted structures (GTV cm)	0.88 (0.54)	0.26 (0.22)	<.0001
Distance to adjusted structures (PTV cm)	0.48 (0.36)	0.19 (0.18)	0.0001

Central+Peripheral: central and peripheral lesions for which all the dose constraints were met. Central_no_: central lesions for which ≥1 dose constraints were not met. Std. dev.: standard deviation. GTV: gross tumor volume. PTC: planning target volume. #: number. cm: centimeter. cc: cubic centimeter. OARs: organs at risk.

The majority of the lesions was in the left or right upper lobes of the lungs for the centrally located lesions and the peripheral lesions. They were consisted of 68.42%, 47.82%, and 65% of the Central, Central_no_, and Peripheral groups of lesions (*p*>0.05).

## Discussion

SABR or SBRT has emerged to become a major treatment approach for early-stage NSCLC or lung metastases with excellent local control in recent years [18], [19]. However, severe toxicities following SABR have been associated with centrally located lesions, which are in close proximity to critical organs [7]–[10]. The reported fatal complications, mostly grade 5 hemoptysis, are usually associated with a high dose delivered per fraction. This is presumably due to excessive radiation dose to the normal structures (Table 6) [7], [8], [10], [20]–[23]. Thus, the clinician is faced with a dilemma in this situation: Lowering radiation dose or compromising target coverage may be associated with a high risk of local recurrence and death from tumor progression; or delivering a high dose of radiation which may lead to potentially fatal treatment related toxicities. This prompted us to search for an optimal dose fractionation schedule that may reliably deliver a high dose to the tumor while respecting the constraints to the surrounding normal tissues. In a previous study, we demonstrated that helical tomotherapy, by virtue of its unique radiation delivery approach, may be the ideal IMRT delivery system when treating central lesions with SABR because of the sharp dose gradient it generates, especially for the 7 Gy×10 fractions schedule [11].

**Table 6:** Grade 5 toxicity reported in the literature following stereotactic body radiotherapy for centrally located lung cancer.

Study	Dose fractionation schedule linked to fatal complications	Cause of death	Incidence of death (%)
Timmerman et al^7^ & Fakiris et al^8^	20 Gy×3 Fr†; 22 Gy×3 Fr	Pneumonia, hemoptysis, or respiratory failure	4/22 (18.2%)
Song et al^10^	12 Gy×4 Fr	Hemoptysis	1/9 (11.1%)
Oshiro et al^20^ *	25 Gy×1 Fr	Hemoptysis	1/21 (4.8%)
Peulen et al^21^ *	11 Gy×3 Fr; 8 Gy×5 Fr; 15 Gy×3 Fr	Hemoptysis	3/11 (27.3%)
Milano et al^22^ *	4 Gy×12 Fr; 5 Gy×10 Fr	Hemoptysis, severe dyspnea, or bronchitis	4/53 (7.5%)
Stauder et al^23^	12 Gy×4 Fr	Bronchial obstruction	1/47 (2.1%)

† Fr: fractions;

* previously irradiated patients.

In the current study, we further characterize the physical and geometric parameters of solitary lung lesions (central & peripheral) from patients for whom adequate target and OAR dose parameters can be satisfied for HT-based SABR. The OAR dose constraints are in line with those used in the RTOG phase II trial (0236) on SBRT for peripheral T1-2N0M0 NSCLC in terms of the isodose effect through the linear quadratic formalism. In RTOG 0236, only 3.6% grade 4 SBRT related toxicity and no grade 5 toxicity were reported [15]. The spinal cord dose was kept lower due to the concern of increased risk for intrafractional motion when delivering SABR in complicated cases, which often takes a long time. However, low dose to the spinal cord is easily achieved in our experience. In challenging cases, we are willing to accept a maximum spinal cord dose of 30 Gy, which are still acceptable based on accepted practice of treating metastases causing spinal cord compression; and QUNTEC recommendations suggesting that 21 Gy delivered in 3 fractions will translated to <1% risk of myelopathy [24]. In addition, SABR/SBRT treatments delivering a mean linear quadratic 2 Gy equivalent dose (EQD_2_) of 36.4 Gy (α/β of 2) as maximum point dose was found to be safe with no occurrence of myelopathy, which also supports our practice of keeping our maximum point dose to slightly <30 Gy when it can be easily achieved [25]. In our limited experience, no toxicity has been encountered in patients who were treated with the current set of dose constraints used (Table 1).

To our best knowledge, this is the first study to establish a set of preliminary, yet clinically applicable dosimetric guidelines to aid the selection of centrally located lesions for HT-based SABR. The 7 Gy×10 fraction schedule was chosen mainly because concerns of significant normal tissue toxicity associated with fractionations schedules of shorter duration when centrally located lesions were treated [7]–[10], [20]–[23]. In addition, the treatment time for delivering a high dose is usually fairly long for helical tomotherapy. As a result, most institutions usually divide fractional doses ≥10 Gy into two consecutive treatments of equal dose with pre-treatment MVCT set up verification before each treatment on a daily basis. This treatment approach appears to be inconvenient and time consuming in a busy clinic. On the contrary, the 10 fraction schedule is not only found to be associated with an excellent toxicity profile with only 1 case of grade 3 pneumonitis out of 43 patients when fairly large tumors were treated, but also delivers a fractional dose that can be delivered in 1 treatment with helical tomotherapy [12]. Thus, this schedule appears to be a very good choice among many well tested treatment schedules when it comes to treating central lesions with helical tomotherapy. The results indicate that large, centrally located lesions that are in close proximity to multiple OARs are difficult to treat without overdosing the OARs if optimal target dose coverage is desired. These lesions tend to be associated with significantly more heterogeneous dose distribution in the PTV, and more dose scatter to the contralateral lung (Tables 2 and 3). Larger central lesions usually are closer to the immediately adjacent critical thoracic structures than central lesions of a smaller size. Thus, demanding a sharper dose gradient to be generated between the PTV's edge and the immediately adjacent OARs. However, they are often surrounded by an increased number of OARs, which greatly limits the entry angle for free entering beams, thus making adequately covering the PTV without depositing a high dose in the immediately adjacent structures impossible.

Because of the limitation on intensity modulation imposed by the increased number of OARs associated with large central lesions, HT-based SABR may not be the most optimal treatment technique if optimal target coverage is desired, even if other dose fractionation schedules are considered. This is evidenced in a study by Baisden *et al* using mostly 3 fraction schedules of various doses, which had to accept less optimal target dose coverage when an OAR is very close to the tumor [26]. In such situations, a high dose delivered through a more protracted course while accounting for tumor shrinkage may be a good alternative to SABR. However, this will need to be further investigated in the future.

The normal lung dose does not seem to be a decisive factor in the feasibility of HT-based SABR. V_20_ is well below 20% for all cases. V_20_ below 20% was previously shown to be associated with only 1 case of maximally grade 3 pneumonitis (2.3%) in a cohort of patients treated with 7 Gy×10 fractions to the GTV [12]. Also, the MLD for the total lung are well below 14 Gy_3_ on average in all cases, which puts them at a low risk for severe radiation pneumonitis as shown in many studies [19], [27]. On the contrary, the increased number of OARs immediately adjacent to the tumor target seems to be the key reason for suboptimal critical structure sparing if adequate PTV dose coverage is to be maintained. As a result, we propose to use tumor size (both tumor diameter and volume), the number of separate critical structures immediately adjacent to the tumor, and the distances of the closest structures to the GTV and PTV as a starting point in selecting patients for HT-based SABR.

There are limitations to our study which need to further investigated. One of them is tumor motion management. HT has been previously demonstrated to be adequate for treating moving targets with a hypofractionated course of radiotherapy [28]. Tumor motion can be addressed with four-dimensional (4D) CT to account for internal tumor motion throughout the entire respiratory cycle; while set up errors can be further reduced with HT compatible immobilization devices [29]. 4-D CT has been adopted in our institution since 2009. However, this is not the most essential issue in our study, which only needs hypothetical targets to carry out the investigation. In addition, the prolonged treatment delivery time of a complex treatment plan associated with HT may be partially resolved with newer technology, such as dynamic jaws and dynamic couch which come with the next generation of HT systems [30]. We recognize that there are other dose fractionation schedules, which may be used when treating centrally located lesions. However, as Table 6 illustrates, fatal complications are more likely to occur with high fractional doses, which prompted us to take a conservative approach in dose fractionation selection which is only limited to schedules for which clinical outcome and toxicity profile were previously reported. This may help clinicians in designing prospective SABR trials treating centrally located tumors in the future.

In conclusion, meeting the OAR dose constraints with adequate PTV target dose coverage criteria is most likely to be accomplished for lung lesions with the following characteristics: GTV≤3.78 cm, or 11.98 cc; PTV≤4.90 cm, or 34.43 cc; ≤2 separate adjacent structures immediately adjacent to the GTV, and the distances of GTV and PTV to the OARs of ≥0.45 cm, and ≥0.21 cm, respectively, when the 7 Gy×10 fractions schedule is delivered with HT. As these are only preliminary findings, they will need to be further validated in a phase I prospective trial evaluating SABR for early stages NSCLC and/or solitary lung metastases.
